# The Combination of Early and Rapid Type I IFN, IL-1α, and IL-1β Production Are Essential Mediators of RNA-Like Adjuvant Driven CD4+ Th1 Responses

**DOI:** 10.1371/journal.pone.0029412

**Published:** 2011-12-19

**Authors:** Rachel F. Madera, Jennifer P. Wang, Daniel H. Libraty

**Affiliations:** Division of Infectious Disease and Immunology, Department of Medicine, University of Massachusetts Medical School, Worcester, Massachusetts, United States of America; University of Cape Town, South Africa

## Abstract

There is a growing need for novel vaccine adjuvants that can provide safe and potent T-helper type 1 (Th1) activity. RNA-like immune response modifiers (IRMs) are candidate T-cell adjuvants that skew acquired immune responses towards a Th1 phenotype. We set out to delineate the essential signaling pathways by which the RNA-like IRMs, resiquimod (R-848) and polyinosinic:polycytidylic acid (poly I:C), augment CD4+ T-helper 1 (Th1) responses. Highly purified murine conventional dendritic cells (cDCs) and conventional CD4+ T-cells were co-cultured in allogeneic and MHC congenic mixed leukocyte reactions. The activation of CD4+ Th1 cells was examined utilizing cells from mice deficient in specific RNA-sensing pattern recognition receptors and signaling mediators. R-848 and poly I:C stimulation of Type I interferon production and signaling in cDCs was essential but not sufficient for driving CD4+ Th1 responses. The early and rapid production of IL-1α and IL-1β was equally critical for the optimal activation of Th1 CD4+ T-cells. R-848 activation of Toll-like receptor 7/MyD88-dependent signaling in cDCs led to a rapid upregulation of pro-IL-1α and pro-IL-1β production compared to poly I:C activation of MyD88-independent signaling pathways. The *in vitro* data show that CD4+ T-cell adjuvant activity of RNA-like IRMs is mediated by a critical combination of early and rapid Type I interferon, IL-1α and IL-1β production. These results provide important insights into the key signaling pathways responsible for RNA-like IRM CD4+ Th1 activation. A better understanding of the critical signaling pathways by which RNA-like IRMs stimulate CD4+ Th1 responses is relevant to the rational design of improved vaccine adjuvants.

## Introduction

Adjuvants are important in eliciting robust protective immune responses from vaccines but many of their underlying mechanisms are yet to be fully elucidated [1]. Vaccine adjuvants mainly target professional antigen-presenting cells (APCs) such as dendritic cells and activate innate immunity through pattern recognition receptor (PRR) pathways [1], [2]. For protection against most viruses and intracellular pathogens, adjuvants that stimulate CD4+ T helper type 1 (Th1) responses are desirable. CD4+ T-cell help is known to be required for optimizing B-cell and CD8+ T-cell responses, and can also provide protection through direct cytotoxic effector functions [3], [4]. Unfortunately, potent CD4+ T-cell adjuvant activity in humans has often been associated with unacceptable toxicity (*e.g.* complete Freund's adjuvant [5]). Therefore, one of the major challenges in adjuvant research has been to gain CD4+ Th1 stimulatory activity while minimizing potential toxicity.

RNA-like immune response modifiers (IRMs) can skew acquired immune responses towards a Th1 phenotype while suppressing Th2 responses [6], [7], [8], [9]. Among these RNA-like IRMs, resiquimod (R-848) and polyinosinic:polycytidylic acid (poly I:C) are being evaluated as T-cell adjuvants for vaccine development [7], [10], [11], [12]. R-848 is a synthetic imidazoquinoline-like molecule that triggers cellular responses via the endosomal Toll-like receptors (TLRs) 7 and 8 and MyD88-dependent signaling [13], [14]. Poly I:C is a synthetic analog of viral dsRNA that activates MyD88-independent immune responses through TLR3/TIR-domain-containing adapter-inducing interferon-β **(**TRIF) and the melanoma differentiation associated protein 5 (MDA5)/Interferon-β promoter stimulator 1 (IPS-1) signaling pathways [15], [16].

These RNA-sensing PRRs and signaling pathways are present in APCs and CD4+ T-cells [17], [18]. RNA-like IRM activation of MyD88-dependent and MyD88-independent signaling pathways can induce a broad range of cell-specific responses, including NF-κB activation, type I interferon (IFN) and pro-inflammatory cytokine production, and co-stimulatory molecule upregulation [6], [9], [17]. The ability of RNA-like adjuvants to stimulate CD4+ Th1 responses likely depends on a combination of key signaling pathways in APCs and CD4+ T-cells. A better understanding of the critical signaling pathways by which RNA-like IRMs stimulate CD4+ Th1 responses will help in the establishment of effective strategies in the generation of rationally designed vaccine adjuvants.

In this paper, we set out to delineate the essential signaling pathways by which the RNA-like IRMs, R-848 and poly I:C, augment CD4+ Th1 responses. Highly purified conventional dendritic cells (cDCs) and conventional CD4+ T-cells were co-cultured in mixed leukocyte reactions (MLRs) in order to evaluate the specific RNA-like adjuvant effects on these central mediators of primary immune responses. We found that R-848 was a more effective CD4+ Th1 adjuvant than poly I:C in isolated cDC/CD4+ T­-cell interactions. Type I IFN production and Type I IFN receptor signaling in cDCs were necessary but not sufficient for the CD4+ T-cell adjuvant activity of the R-848. Early and rapid IL-1α production and IL-1β secretion from cDCs were also necessary for the CD4+ Th1 adjuvant properties of R-848. Moreover, addition and blocking of these essential cytokines affect RNA-like IRM stimulated CD4+ Th1 responses. Our results provide important insights into the key signaling pathways responsible for RNA-like IRM CD4+ T-cell activation, and will help in the rational design of improved vaccine adjuvants.

## Materials and Methods

### Reagents

Resiquimod (R-848) was purchased from 3 M Pharmaceuticals (St. Paul, MN) and poly I:C was purchased from Alexis Biochemicals (San Diego, CA). Purified FITC-anti-mouse CD11c, PE-anti-mouse CD11b, PerCP-anti-mouse-CD45R, FITC-anti-mouse CD4, PerCPCy5.5-anti-mouse CD4, PerCPCy5.5-anti-mouse CD8, PE-anti-mouse TCRβ, Alexa 700-anti-mouse tumor necrosis factor-α (TNF-α) and Allophycocyanin-anti-mouse IFN-γ were purchased from BD Biosciences (San Jose, CA). Purified Pacific Blue-anti-mouse CD3, and PE-Cy7-anti-mouse DX5 were purchased from eBiosciences (San Diego, CA). For the add-in and blocking experiments, the following reagents were used: universal Type I IFN (PBL Biomedical Laboratories, Piscataway, NJ), mouse rIL-1α, rIL-1β and soluble IL-1 receptor antagonist (sIL-1Ra) (R & D Systems, Minneapolis, MN), Anakinra™ (sIL-1R antagonist) (Amgen, Thousand Oaks, CA), anti-mouse IFN α/β receptor IgG_1_ (anti-IFNAR) (Leinco, St. Louis, MO), and purified mouse IgG_1_ isotype control (BD Biosciences).

### Mice

C57BL/6J (B6), BALB/cJ and B6.C-H2d/bByJ (B6.H2d) mice were purchased from The Jackson Laboratory (Maine, USA). MyD88^−/−^, TLR7^−/−^, IL-1R^−/−^ and type I IFN receptor^−/−^ (IFNAR^−/−^) mice were provided by Drs. R. Finberg and K. Fitzgerald (University of Massachusetts Medical School, Worcester, MA) [19], [20], [21]. IL-1α^−/−^, IL-1β^−/−^ and IL-1αβ^−/−^ mice were provided by Drs. K. Rock and H. Kono (University of Massachusetts Medical School) [22], [23]. The genetically modified knockout mice were backcrossed eight or more generations onto the B6 background and were then intercrossed to obtain the knockout genotypes. All animal procedures were conducted in strict accordance with the recommendations in the Guide for the Care and Use of Laboratory Animals of the National Institutes of Health. The protocol was approved by the University of Massachusetts Medical School Animal Care and Use Committee (Protocol A-1884).

### cDCs and CD4+ T cells

Bone-marrow derived dendritic cells were prepared as described previously [24]. In brief, bone marrow cells were cultured in RPMI supplemented with 10% FBS, 50 µM 2-mercaptoethanol and 20 ng/ml recombinant *fms*-related tyrosine kinase 3 ligand (rFlt3L, R&D Systems) for 7 days. Bone-marrow derived dendritic cells were scraped off the culture dish, washed with PBS and then stained with fluorochrome-conjugated antibodies for fluorescence-activated cell sorting (FACS). cDCs (CD11c^+^CD11b^+^CD45R^−/lo^) were isolated by sorting on a BD FACSAria flow cytometer (BD Biosciences). Conventional CD4+ T-cells were isolated from mice splenocytes by magnetic bead enrichment for CD4+ T-cells (MACS, Miltenyi Biotec, Auburn, CA) followed by flow cytometry sorting for the CD3^+^CD4^+^CD8^−^DX5^−^TCRβ^+^ lymphocytes. Analysis of the cDC and CD4+ T-cells after FACS consistently demonstrated ≥99% purity. The sorted CD4+ T-cells were at least 80% CD62L^hi^ indicating mostly naïve CD4+ T-cells. Cell culture supernatants and cellular RNA were collected at the indicated time points for ELISA and RT-PCR analyses.

### Mixed Leukocyte Reactions, (MLR)

FACS-sorted cDCs and CD4+ T cells were co-cultured for six days in allogeneic and MHC congenic MLRs. Initial experiments were done using cells from wild-type B6 (*H2^b^*) and BALB/c (*H2^d^*) mice to have an allogeneic response, and subsequently switched to using MHC congenic *H2^d^* mice on the B6 background, B6.C-H2d/bByJ (B6.H2d). No apparent differences between the allogeneic or MHC congenic systems were observed. The murine TLR7 agonist, R-848, and the TLR3 and MDA5 agonist, poly I:C, were added to MLRs at final concentrations of 20 µM and 100 µg/ml, respectively. These agonist concentrations produced maximal T-cell responses in preliminary experiments. After six days, cell-free culture supernatants were collected for ELISA and cells were collected for intracellular cytokine staining (ICS).

### Intracellular cytokine staining

IFN-γ and TNF-α-secreting CD4+ T-cells were quantified by ICS assay. Briefly, cells from MLRs were washed with media then cultured for 5 to 6 hours without restimulation in the presence of Brefeldin A (BD Biosciences, San Jose, CA). Cells were stained with surface antibodies, and permeabilized with Cytofix/Cytoperm (BD Biosciences, San Jose, CA) before intracellular staining with antibodies and fixation. CD4+ T-cells were analyzed using a BD FACSAria cytometer. LIVE/DEAD® Fixable Dead Cell Stain Kit (Invitrogen, Carlsbad, CA) was used to exclude nonviable cells from analysis.

### ELISA

Cell culture supernatants were collected and analyzed for mouse IFN-γ, IL-1α and IL-1β by ELISA according to the manufacturer's protocol (BD Biosciences, San Jose, CA). The ELISA plates were developed using 3,3′,5,5′-tetramethylbenzidine (KPL, Gaithersburg, MD), and the reactions were stopped with 2N sulfuric acid. Optical densities were read at 450 nm using a Spectra MAX microplate reader and analyzed with Softmax® Pro 5 Software (Molecular Devices, Sunnyvale, CA).

### Quantitative RT-PCR

IL-1α, IL-1β and IFN-β mRNA expression were determined using RNeasy Plus Mini Kit for RNA extraction (Qiagen, Valencia, CA) and TaqMan RNA-to-C_T_ 1-step kit for RT-PCR (Applied Biosystems, Carlsbad, CA). All primers and probes used were TaqMan Pre-Developed Assay Reagents for Gene Expression (Applied Biosystems, Carlsbad, CA). Levels of gene expression were normalized to β-actin for each sample. Fold increase in gene expression was determined using the ΔΔC_T_ method. Samples were run in triplicates and the results are presented as linear-fold changes in gene expression.

### Addition of IL-1 and blockade of type I IFN and IL-1α/β signaling

Poly I:C stimulated MHC congenic MLRs had mouse rIL-1α and rIL-1β (125 pg/ml each) added. To block endogenous type I IFN and IL-1 signaling, anti-mouse IFN α/β receptor IgG_1_ (5 µg/ml) and Anakinra™ (10 µg/ml) or recombinant mouse sIL-1Ra (1 µg/ml) were added to R-848 stimulated MHC congenic MLRs. Purified mouse IgG_1_ isotype antibody (5 µg/ml) was used as a control. After six days, cell-free culture supernatants were collected for ELISA and cells were collected for ICS, as previously described.

### Statistical analysis

Statistical analysis was done using GraphPad Prism Software version 5.00 (GraphPad, San Diego, CA). Comparisons between two groups were performed using paired or unpaired Student's t-test, as appropriate. Comparisons among >2 groups were performed using analysis-of-variance (ANOVA). A difference was considered significant if the p-value was <0.05.

## Results

### R-848>poly I:C augmented alloreactive CD4+ Th1 responses in a cDC/CD4+ T-cell MLR

**Figure 1:**
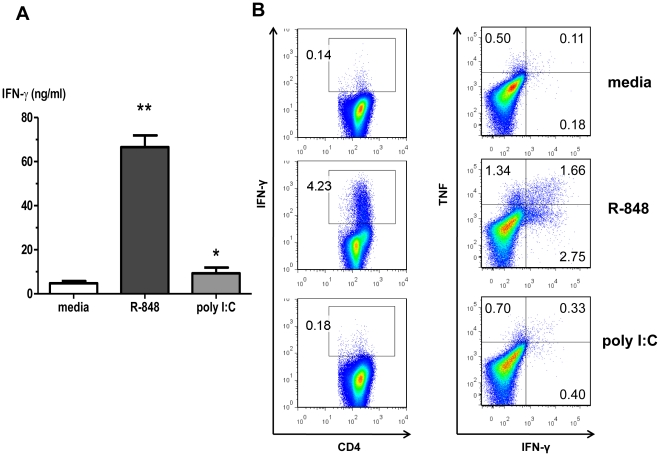
R-848>poly I:C induces IFN-γ+ and TNF-α+ alloreactive CD4+ T-cells in a MLR. Highly purified bone-marrow derived conventional dendritic cells (cDCs)(CD11c^+^CD11b^+^CD45R^−/lo^) and conventional CD4+ T-cells (CD3^+^ CD4^+^CD8^−^TCRb^+^DX5^−^) were co-cultured for 6 d in the presence or absence of RNA-like IRMs. (A) Cell culture supernatant IFN-γ levels. Values are mean ± SEM (n = 3), **R-848 p<0.0001 compared to media control; *poly I:C p = 0.0343 compared to media control. (B) ICS staining for IFN-γ and TNF-α producing conventional CD4+ T-cells (representative of three independent experiments shown). Values shown are the % of CD3^+^CD4^+^ T-cells producing IFN-γ (left column) and % IFN-γ^+^TNF-α^−^, IFN-γ^−^TNF-α^+^, or IFN-γ^+^TNF-α^+^ (right column).

We utilized a MHC congenic MLR to investigate the essential signaling pathways by which RNA-like IRMs stimulate CD4+ Th1 responses. Highly purified murine cDCs and highly purified MHC congenic conventional CD4+ T-cells were co-cultured for six days in the presence or absence of RNA-like IRMs. The cDCs and CD4+ T-cells were sorted by flow cytometry to high purity in order to eliminate the potential effects of bystander cells (*e.g.* natural killer cells, γδ T-cells). Alloreactive CD4+ T-cell cytokine production was measured in cell culture supernatants and by ICS. At maximal doses, the murine TLR7 agonist R-848 was a strong stimulator of alloreactive IFN-γ-producing CD4+ T-cells; whereas, the TLR3 and MDA5 agonist poly I:C was less potent in this system (Figures 1A&B). Nearly all R-848 or poly I:C induced IFN-γ+ CD4+ T-cells also produced TNF-α (Figure 1B) and IL-2 (data not shown). From here on, RNA-like IRM augmentation of CD4+ Th1 responses are reported as IFN-γ expression and production. The data indicated that RNA-like IRM activation of MyD88-dependent signaling pathways (R-848) stimulated CD4+ Th1 responses better than MyD88-independent signaling pathways (poly I:C) in isolated cDC/CD4+ T-cell interactions.

### Type I IFN signaling in cDCs was necessary for RNA-like IRMs to stimulate CD4+ Th1 responses in a cDC/CD4+ T-cell MLR

**Figure 2:**
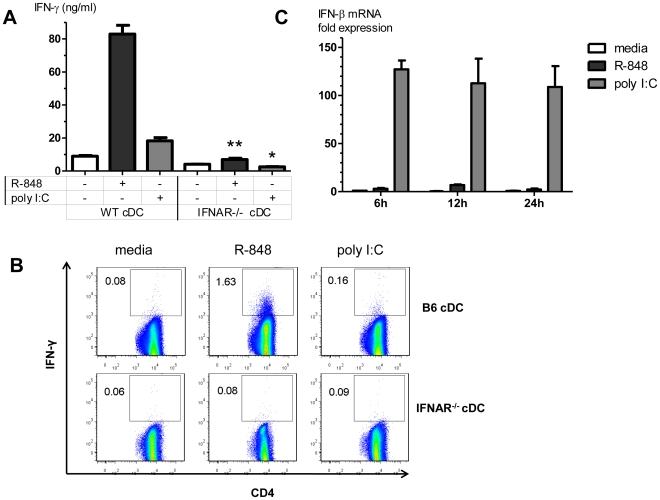
Type I IFN is essential for RNA-like IRMs to stimulate CD4+ Th1 responses in a cDC/CD4+ T-cell MLR. (A) IFN-γ levels in cell culture supernatants in MHC congenic MLRs utilizing B6 or IFNAR^−/−^ cDCs. Values are mean ± SEM (n = 3). ** p = 0.0008 compared to R-848 stimulated B6 cDC MLR, * p = 0.0037 compared to poly I:C stimulated B6 cDC MLR. (B) ICS staining for IFN-γ producing conventional CD3^+^CD4^+^ T-cells in MHC congenic MLRs utilizing B6 or IFNAR^−/−^ cDCs (representative of three independent experiments shown). Values shown are the % of IFN-γ^+^ conventional CD3^+^CD4^+^ T-cells. (C) cDCs were stimulated with R-848, poly I:C, or media (unstimulated control), and cellular RNA was collected at the indicated time points. IFN-β mRNA levels were measured by qRT-PCR, as described in the Methods section. Bars represent fold-change in IFN-β mRNA relative to unstimulated control at the 6 h time point (mean ± SEM, n = 3).

RNA-like IRMs and RNA-sensing PRRs induce Type I IFN production and signaling in cDCs [7], [11], and Type I IFN can drive DC maturation and activation [25]. R-848 stimulated alloreactive CD4+ T-cell IFN-γ responses were abolished in IFNAR^−/−^ cDC/B6.H2d CD4+ T-cell MLR compared to wild-type B6 cDC/B6.H2d CD4+ T-cell MLR (Figures 2A&B). The small increase in alloreactive CD4+ T-cell IFN-γ responses induced by poly I:C was also abrogated when utilizing IFNAR^−/−^ cDCs. Type I IFN signaling in cDCs was essential for the ability of R-848 and poly I:C to augment alloreactive CD4+ T-cell IFN-γ production in isolated cDC/CD4+ T-cell interactions. However, R-848 induced much lower IFN-β mRNA levels in cDCs compared to poly I:C (Figure 2C), and yet stimulated much higher CD4+ T-cell IFN-γ responses compared to poly I:C. This suggested that Type I IFN production and signaling in cDCs was essential but not sufficient for optimal RNA-like IRM stimulation of CD4+ Th1 responses.

### R-848 stimulation of CD4+ Th1 responses was dependent on MyD88-mediated signaling in cDCs and conventional CD4+ T-cells

**Figure 3:**
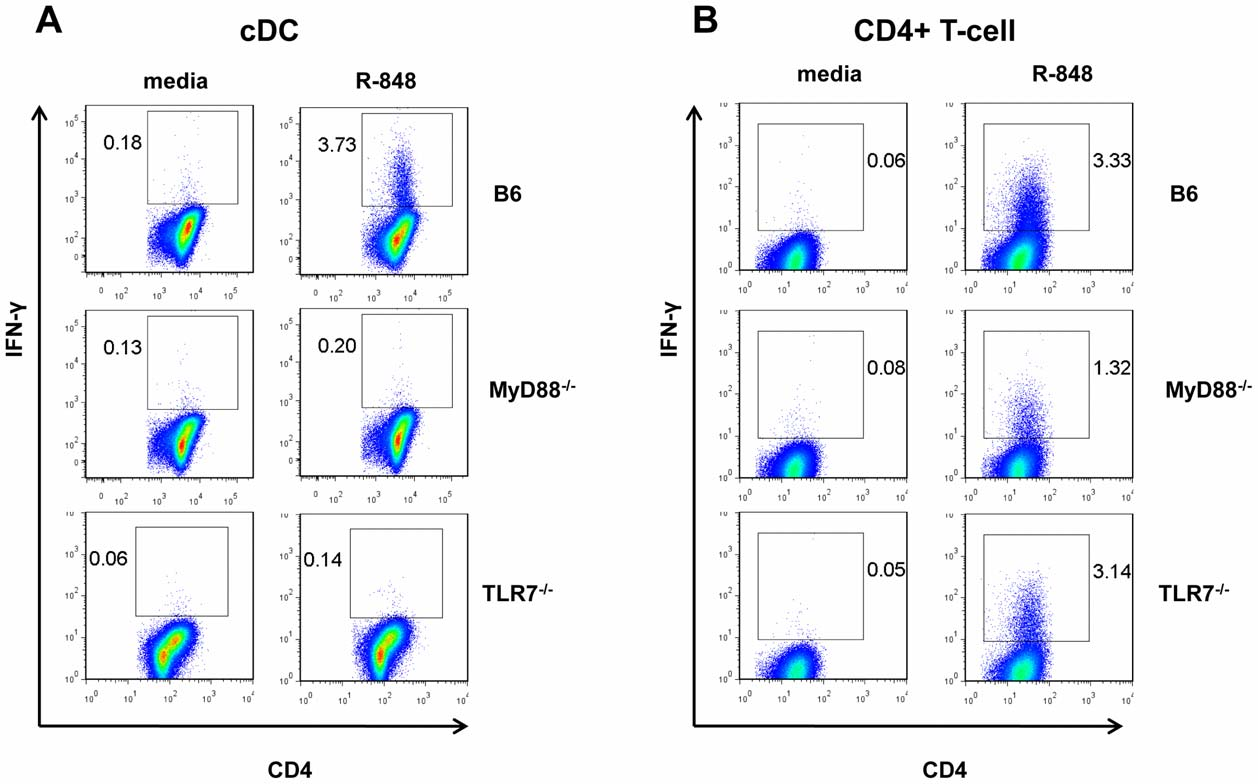
TLR7 signaling in cDCs, and TLR7-independent/MyD88-mediated signaling in CD4+ T-cells, are essential for R-848 induced CD4+ Th1 responses. (A) ICS staining for IFN-γ producing CD4+ T-cells in MHC congenic MLRs utilizing B6, MyD88^−/−^, or TLR7^−/−^ cDCs (representative of two independent experiment shown) (B) ICS staining for IFN-γ producing CD4+ T-cells in MHC congenic MLRs utilizing B6, MyD88^−/−^, or TLR7^−/−^ CD4+ T-cells (representative of two independent experiments shown). Values shown are the % of IFN-γ^+^ conventional CD3^+^CD4^+^ T-cells.

R-848 activates murine TLR7/MyD88-dependent signaling [26] and R-848 has been reported to have direct effects on cDCs and CD4+ T-cells [18], [27], [28]. We therefore examined the role of MyD88-dependent signaling pathways in cDCs and CD4+ T-cells during R-848 stimulation of alloreactive CD4+ Th1 responses. Not surprisingly, R-848-induced alloreactive CD4+ T-cell IFN-γ production was entirely abrogated when MyD88^−/−^ or TLR7^−/−^ cDCs were used in MHC congenic MLRs (Figure 3A). However, R-848-induced alloreactive CD4+ T-cell IFN-γ production was also fully abrogated when MyD88^−/−^ conventional CD4+ T-cells were used in MLRs. This MyD88-dependence in conventional CD4+ T-cells was not mediated through TLR7 or IL-18 receptor signaling (Figure 3B and data not shown). The data indicated that R-848 activation of TLR7/MyD88-dependent signaling in cDCs was critical for augmenting alloreactive CD4+ Th1 responses, and that TLR7-independent/MyD88-dependent signaling in CD4+ T-cells was equally critical.

### cDC IL-1α and IL-1β production, and IL-1R-mediated signaling in cDCs and conventional CD4+ T-cells, were essential for R-848 stimulation of CD4+ Th1 responses

**Figure 4:**
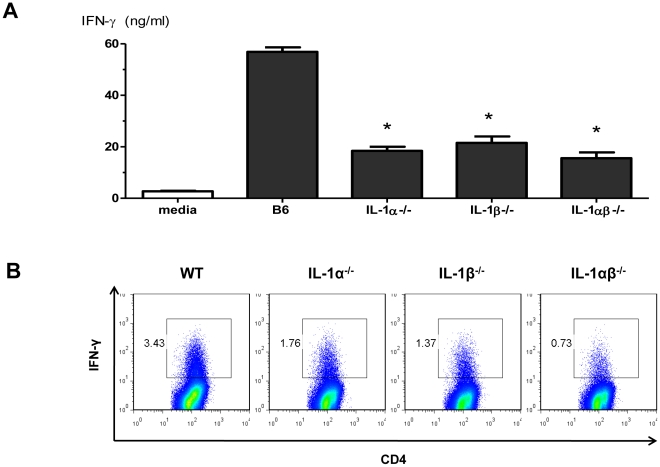
IL- 1α and IL-1β are essential for R-848 stimulation of CD4+ Th1 responses. (A) Cell culture supernatant IFN-γ levels (day 6) in MHC congenic MLRs utilizing B6, IL-1α^−/−^, IL-1β^−/−^ or IL-1αβ^−/−^ cDCs. Values are mean ± SEM (n = 2). *p<0.05 compared to R-848 stimulated B6 cDC MLR. (B) ICS staining for IFN-γ producing CD4+ T-cells in MHC congenic MLRs utilizing B6, IL-1α^−/−^, IL-1β^−/−^ or IL-1αβ^−/−^ cDCs (representative of two independent experiments shown). Values shown are the % of IFN-γ^+^ conventional CD3^+^CD4^+^ T-cells.

We next explored the role of IL-1R/MyD88 signaling in R-848 stimulation of CD4+ Th1 responses. When IL-1R^−/−^ cDCs were used in the MHC congenic MLRs, R-848-induced alloreactive CD4+ T-cell IFN-γ production was 45±9% lower than when wild-type cDCs were used (mean±SEM, *n* = 3, p = 0.03). Similarly, when IL-1R^−/−^ CD4+ T-cells were used in MHC congenic MLRs, R-848-induced alloreactive CD4+ T-cell IFN-γ production was 55±5% lower than wild-type CD4+ T-cells (mean±SEM, *n* = 3, p = 0.01). The observed effect of IL-1R-mediated signaling on R-848 stimulation of CD4+ Th1 responses in both cDC and CD4+ T-cells prompted us to examine the roles of cDC IL-1α and IL-1β production. R-848-induced alloreactive CD4+ T-cell IFN-γ production was nearly completely abrogated when IL-1α^−/−^, IL-1β^−/−^, or IL-1αβ^−/−^ cDCs were used in the MLRs (Figures 4A&B). IL-1α and IL-1β had largely non-redundant roles in driving the alloreactive CD4+ Th1 responses to R-848 in this system. Collectively, our data suggest that the combination of Type I IFN, IL-1α, and IL-1β are necessary for the optimal stimulation of CD4+ Th1 responses by RNA-like IRMs.

### R-848 rapidly increases cDC production of pro-IL-1α and pro-IL-1β mRNA and protein

**Figure 5:**
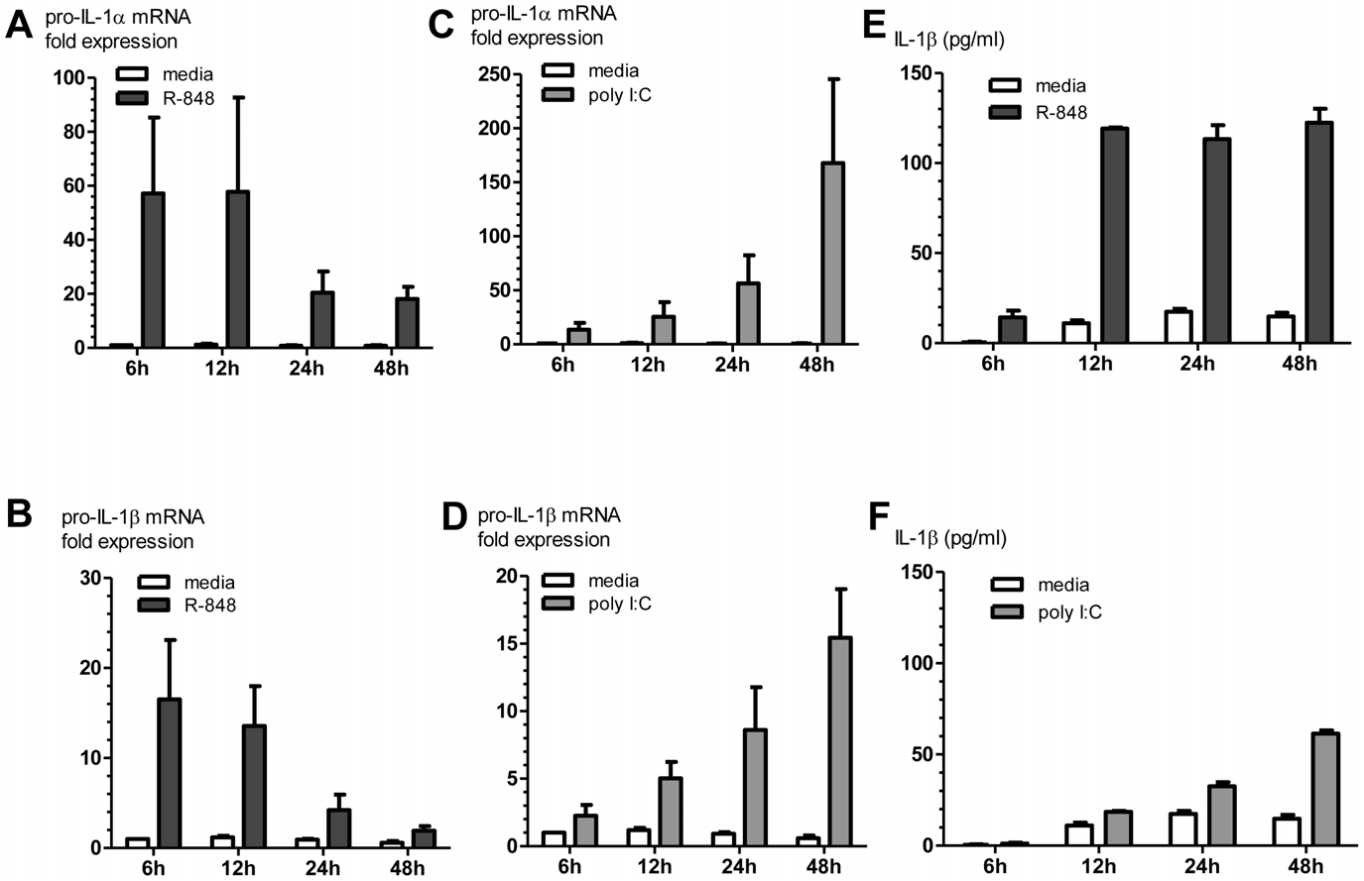
R-848 induces a rapid increase in pro-IL- 1α and pro-IL-1β mRNA expression and protein production in cDCs. cDCs were stimulated with R-848, poly I:C, or media (unstimulated control), and cellular RNA and cell culture supernatants were collected at the indicated time points. Pro-IL- 1α (A&B) and pro-IL-1β (C&D) mRNA levels were measured by qRT-PCR, as described in the Methods section. Bars represent fold-change in pro-IL- 1α or pro-IL-1β mRNA relative to unstimulated control at the 6 h time point (mean ± SEM, n = 3). (E&F) IL-1β protein levels (precursor and mature forms) were measured in cell culture supernatants by ELISA (mean ± SEM, n = 2).

R-848 stimulation of cDCs produced a rapid upregulation of pro-IL-1α and pro-IL-1β mRNA (Figures 5A&B); whereas, poly I:C stimulation of cDCs produced a slower and more gradual increase in pro-IL-1α and pro-IL-1β mRNA expression (Figures 5C&D). Biologically active IL-1α (precursor and mature IL-1α) is primarily intracellular and membrane-associated, while biologically active IL-1α (mature IL-1β) is primarily secreted [29]. Comparable levels of IL-1α protein (precursor and mature forms) were measured in the cell lysates from R-848 and poly I:C-stimulated cDCs at early time points (data not shown). R-848 and extracellular ATP stimulation of cDCs rapidly induced higher levels of secreted mature IL-1β protein in cell-free culture supernatants compared to poly I:C and extracellular ATP (Figures 5E&F, Figure S1).

**Figure 6:**
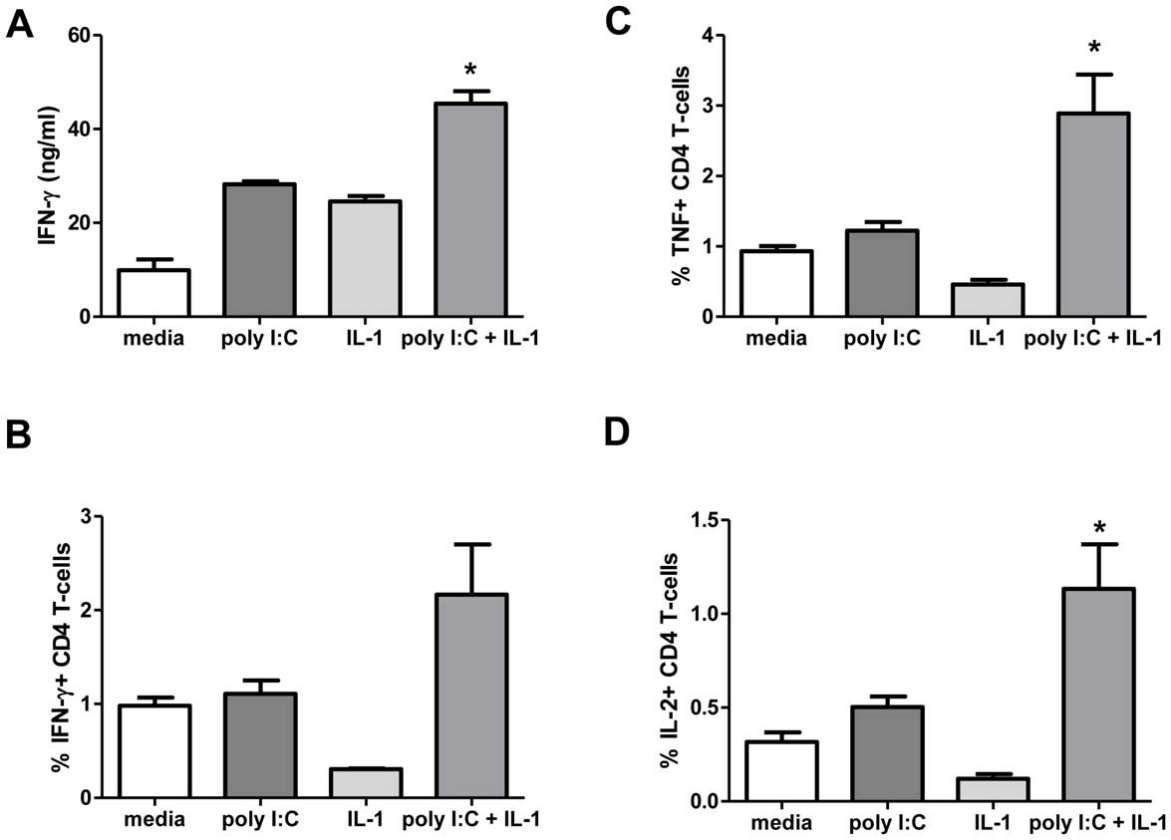
The addition of rIL- 1α and rIL-1β increases poly I:C-induced CD4+ Th1 responses in a cDC/CD4+ T-cell MLR. (A) Cell culture supernatant IFN-γ levels in poly I:C stimulated MHC congenic MLR with mouse rIL- 1α and rIL-1β. ICS staining for IFN-γ (B), TNF-α (C) and IL-2 (D) producing conventional CD4+ T-cells. Values are mean ± SEM (n = 3) (representative of two independent experiments shown). *p<0.05 compared to media control.

When 200 U/ml type I IFN and 125 pg/ml each of rIL-1α and rIL-1β were added to a MHC congenic cDC/CD4+ MLR, type I IFN and IL-1 were sufficient to induce a three-fold increase in alloreactive CD4+ T-cell IFN-γ production and was comparable to poly I:C but not R-848 (data not shown). Poly I:C induced rapid and sustained Type I IFN production (Figure 2C), but induced pro-IL-1α and pro-IL-1β at later time points compared to R-848 (Figure 5). To further examine the early roles of IL-1α and IL-1β, we added mouse rIL-1α and rIL-1β at the same time as poly I:C in MHC congenic MLRs. Addition of IL-1α and IL-1β increased poly I:C induced cytokine-producing (IFN-γ, TNF-α and IL-2) CD4+ T-cells (Figure 6). The data indicated that early IL-1α and IL-1β production and signaling are essential for the robust RNA-like IRM (poly I:C and R-848) stimulation of CD4+ Th1 responses.

### Inhibition of IL-1 and Type I IFN signaling abrogated R-848 stimulation of CD4+ Th1 responses

**Figure 7:**
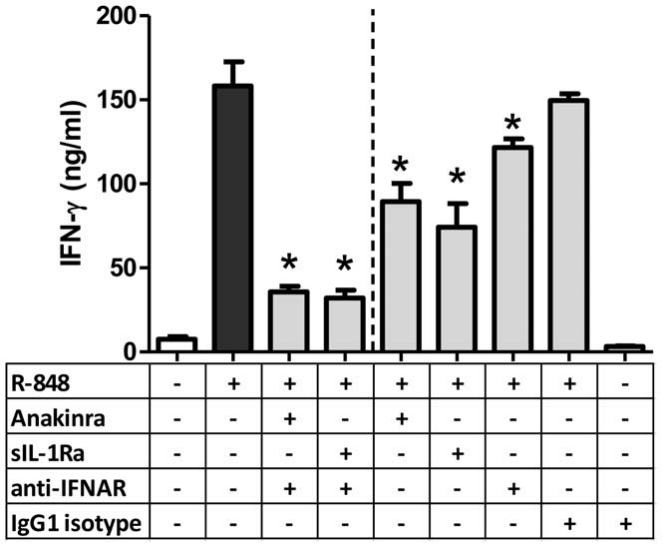
Inhibition of Type I IFN, IL- 1α and IL-1β signaling decreases R-848-induced CD4+ Th1 responses in a cDC/CD4+ T-cell MLR. Cell culture supernatant IFN-γ levels (day 6) in MHC congenic MLRs with Type I IFN and IL-1 signaling blocked by anti-mouse IFN α/β receptor, Anakinra™ and sIL-1Ra. Compared to a R-848 stimulated MHC congenic MLR, decreased CD4+ Th1 responses were observed in MLRs with blocked Type I IFN, IL-1α and IL- 1β signaling. Blocking of either Type I IFN or IL-1α/β signaling also decreased cell culture supernatant IFN-γ levels. Values are mean ± SEM (n = 6). *p<0.05 compared to R-848 stimulated MLR.

To further assess R-848-induced CD4+ Th1 responses, a natural inhibitor of IL-1 signaling, sIL-1Ra, and its synthetic form, Anakinra™, were added to R-848 stimulated MHC congenic MLRs. To inhibit Type I IFN signaling, antibodies against the IFN α/β receptor were also added. Inhibition of both IL-1 and Type I IFN signaling diminished the robust R-848 induced CD4+ Th1 responses (Figure 7). Inhibition of either IL-1 or Type I IFN signaling also reduced R-848 induced CD4+ Th1 responses but to a lesser degree (Figure 7). Taken together, Type I IFN production and signaling by itself was not sufficient to drive RNA-like IRM stimulated CD4+ Th1 responses. The combined actions of Type I IFN, IL-1α, and IL-1β were essential for the optimal activation of alloreactive CD4+ Th1 responses in cDC/conventional CD4+ T-cell interactions.

## Discussion

There is a growing need for novel vaccine adjuvants with potent T-cell immune stimulatory effects. In this paper, we investigated the critical cellular signaling pathways used by RNA-like IRMs (R-848 and poly I:C) to stimulate CD4+ Th1 responses during alloreactive cDC/CD4+ T-cell interactions. RNA-like IRM stimulation of Type I IFN production and signaling was essential but not sufficient for driving CD4+ Th1 responses. The rapid and early production of IL-1α and IL-1β was equally critical for the optimal activation of Th1 CD4+ T-cells. In cDCs, R-848 activation of TLR7/MyD88-dependent signaling led to a rapid upregulation of pro-IL-1α and pro-IL-1β production compared to poly I:C activation of MyD88-independent signaling pathways. R-848 stimulated the early production and secretion of mature IL-1β from cDCs and augmented alloreactive CD4+ Th1 responses to a greater degree than poly I:C. Our data suggests that the CD4+ T-cell adjuvant activity of RNA-like IRMs is mediated by a critical combination of rapid Type I IFN, IL-1α and mature IL-1β production.

The RNA-like IRMs, R-848 and poly I:C, are well-known inducers of Type I IFN in many cell types and have been shown to be potent CD4+ T-cell adjuvants [8], [11], [12], [30]. Longhi *et al.* [7] primed and boosted mice with a dendritic cell targeted HIV gag protein vaccine and TLR agonists. They found poly I:C to be the most effective inducer of Type I IFN and the superior adjuvant to elicit CD4+ T cell immunity. Antibody blocking or deletion of Type I IFN receptor markedly reduced DC maturation, T-cell proliferation, and the development of adaptive Th1 immunity in response to the HIV gag protein. Ichinohe et al. [31] also investigated the mucosal adjuvant effect of poly I:C by intranasal co-administration with inactivated influenza virus HA vaccines and observed cross-protection against heterologous infection. The authors observed upregulated expression of Type I IFN, and Th1/Th2 cytokines following the administration of HA vaccine with poly I:C. Vasilakos *et al.* [8] demonstrated that mice immunized with chicken ovalbumin and R-848 induced high levels of Type I IFN, and neutralizing antibodies to Type I IFN inhibited ovalbumin-specific CD4+ Th1 responses. Similarly, in isolated cDC/CD4+ T-cell interactions, we found that Type I IFN production and signaling was necessary for the activation of Th1 alloreactive CD4+ T-cells by RNA-like IRMs. However, poly I:C was a more potent inducer of Type I IFN in highly purified cDCs compared to R-848, yet it was less potent at stimulating alloreactive CD4+ Th1 responses. This suggested that Type I IFN production and signaling in cDCs was indeed essential but not sufficient for RNA-like IRMs to augment CD4+ Th1 responses in isolated alloreactive cDC/CD4+ T-cell interactions.

We found that the ability of R-848 to augment alloreactive CD4+ Th1 responses in a cDC/CD4+ T-cell MLR was also dependent on the early and rapid production of functional IL-1α and IL-1β from cDCs. MyD88-dependent IL-1R mediated signaling in both cDCs and CD4+ T-cells was important in mediating the CD4+ Th1 stimulatory effect of R-848. IL-1 stimulates cDC maturation and activation [32], [33] and can act directly on CD4+ T-cells to enhance their differentiation and cytokine production [34].

The Th1 adjuvant effects of IL-1 have been previously reported in *in vivo* studies. In the pigeon cytochrome C (PCC) adoptive transfer studies of Ben-Sasson et al. [34], mice recipients were immunized with PCC peptide together with proinflammatory cytokines (administered through a miniosmotic pump). Among the series of cytokines tested, including TNFα, IL-1, IL-6, IL-18 and IL-33, only IL-1 augmented primary immune responses. IL-1α and IL-1β displayed similar potency [34]. In addition to the IL-1 adjuvant effect during antigen priming, both the time and the concentration of IL-1 administration are critical. Staruch et al. [35] have shown that the maximum IL-1 adjuvant effect was 2 hours after the priming dose of bovine serum albumin (BSA) protein antigen. These authors have also observed dose-dependent enhancement of CD4+ T-cell responses to BSA. Similarly, in our MHC congenic MLR system, we found that the early addition of IL-1 conferred increased adjuvant effects of poly I:C.

The observed comparable decreases in IFN-γ producing CD4+ T cells when utilizing IL-1α^−/−^, IL-1β^−/−^ and IL-1αβ^−/−^ cDCs in the MLRs suggested that IL-1α and IL-1β have largely non-redundant roles in stimulating alloreactive CD4+ Th1 responses. Though IL-1α and IL-1β both bind and activate IL-1R [36], differences in their localization might explain their apparent non-redundant effects [29], [37], [38]. Biologically active IL-1α (precursor and mature forms) is predominantly cell- and membrane-associated, and likely acts in an autocrine or even intracrine manner in antigen-presenting cells [29]. By contrast, pro-IL-1β is biologically inactive and has different posttranslational processing requirements than pro-IL-1α. Caspase-1-containing inflammasomes (*e.g.* NLRP3 inflammasome) cleave pro-IL-1β into the biologically active mature form which is secreted into the extracellular environment in a non-classical manner [37], [39]. Thus, mature IL-1β likely acts predominantly in a paracrine or endocrine manner, activating IL-1R signaling in effector cells such as CD4+ T-cells. Other studies conducted on IL-1α and IL-1β-deficient mice have also shown non-redundant roles for these cytokines in immune responses and disease pathogenesis. IL-1β, but not IL-1α, was required for antigen-specific T-cell activation in delayed-type hypersensitivity responses [40] and in T-cell dependent antibody production against sheep red blood cells [41], [42]. IL-1α, but not IL-1β, modulated the antiviral and immunoregulatory activities of IFN-γ [42], and was necessary for cell- and tissue-injury induced sterile inflammatory responses [43]. Further studies are needed to discriminate the potential non-redundant roles of IL-1α and IL-1β in the RNA-like adjuvant augmentation of CD4+ Th1 responses.

In cDCs, R-848 induced less Type I IFN but early and more robust IL-1α and IL-1β production than poly I:C. R-848 was also a more potent CD4+ T-cell adjuvant compared to poly I:C in the cDC/CD4+ T-cell MHC congenic MLR system. However, poly I:C has also been shown to be a potent CD4+ T-cell adjuvant *in vivo* [6], [7], [31]. When administered as an adjuvant in mice, the main source of poly I:C-induced Type I IFN has been reported to be non-hematopoietic cells [7]. In addition, poly I:C has been shown to induce mRNA expression [44] and protein production of IL-1 both *in vitro* [45], [46] and *in vivo* [45], [47], [48]. In our isolated cDC/CD4+ T-cell MHC congenic MLR, poly I:C appears unable to stimulate enough Type I IFN and IL-1. The increase in CD4+ Th1 responses with exogenous rIL-1 in poly I:C stimulated MHC congenic MLRs supports this hypothesis. Furthermore, increased Type I IFN production has been shown to inhibit IL-1 production by inhibiting inflammasome function and inducing regulatory IL-10 [49]. The delayed IL-1 production observed in poly I:C stimulated MHC congenic MLRs may be an effect of the robust poly I:C-induced type I IFN in the isolated cDC/CD4+ T-cell MLR system. The essential roles of these cytokines were also evident in the abrogation of CD4+ Th1 responses upon Type I IFN and IL-1 signaling blockade in R-848 stimulated MHC congenic MLRs. The observed differences in the cDC production of type I IFN, IL-1α and IL-1β between R-848 and poly I:C indicate complex signaling cascades for the initiation of CD4+ Th1 responses. Elucidation and a better understanding of these mechanisms would offer further possibilities for the use of these RNA-like IRMs as adjuvants.

Finally, we observed that the ability of R-848 to augment alloreactive CD4+ Th1 responses was nearly completely dependent on MyD88-mediated signaling in the CD4+ T-cells. IL-1R mediated signaling could only explain part of this profound CD4+ T-cell MyD88 signaling dependence. The isolated removal of CD4+ T-cell TLR7 or IL-18 receptor signaling did not affect R-848 induced alloreactive CD4+ Th1 responses. It is possible that there is overlap and a partial redundancy of the signaling effects in CD4+ T-cells among IL-1R, IL-18R, and TLR7/MyD88 dependent pathways. It may also be possible that other MyD88-dependent signaling pathways in CD4+ T-cells, yet to be identified, are involved in priming and activation [50]. A better understanding of the adjuvant mechanisms that promote CD4+ Th1 responses will be important for the rational design of new vaccine strategies. RNA-like IRMs are effective CD4+ Th1 adjuvants and their ability to induce Type I IFN was previously shown to be critical for their T-cell stimulatory activity. In MLRs utilizing two specific cell types, cDCs and conventional CD4+ T-cells, we have shown that early and rapid IL-1α and mature IL-1β production are equally critical. Together, these results show that the CD4+ T-cell adjuvant activity of RNA-like IRMs involves a complex interplay of critical PRR and innate immune signaling pathways in different cells.

## Supporting Information

Figure S1 **R-848 and extracellular ATP stimulates early mature IL-1β secretion from cDCs.** Non-primed DCs were stimulated for 6 h with R-848 or poly I:C with extracellular ATP (5 mM). As a positive control, cDCs were primed with lipopolysaccharide (200 ng/ml) for 3 hours prior to stimulation with nigericin (10 µM). Mature IL-1β protein was detected by Western blot in cell culture supernatants. Data shown is representative of three independent experiments.
(TIF)
