# Supplementary figures and images for: The Combination of Early and Rapid Type I IFN, IL-1α, and IL-1β Production Are Essential Mediators of RNA-Like Adjuvant Driven CD4+ Th1 Responses
Source: PLoS One. 2011 Dec 19;6(12):e29412. doi: 10.1371/journal.pone.0029412 (PMC3242790; doi:10.1371/journal.pone.0029412)

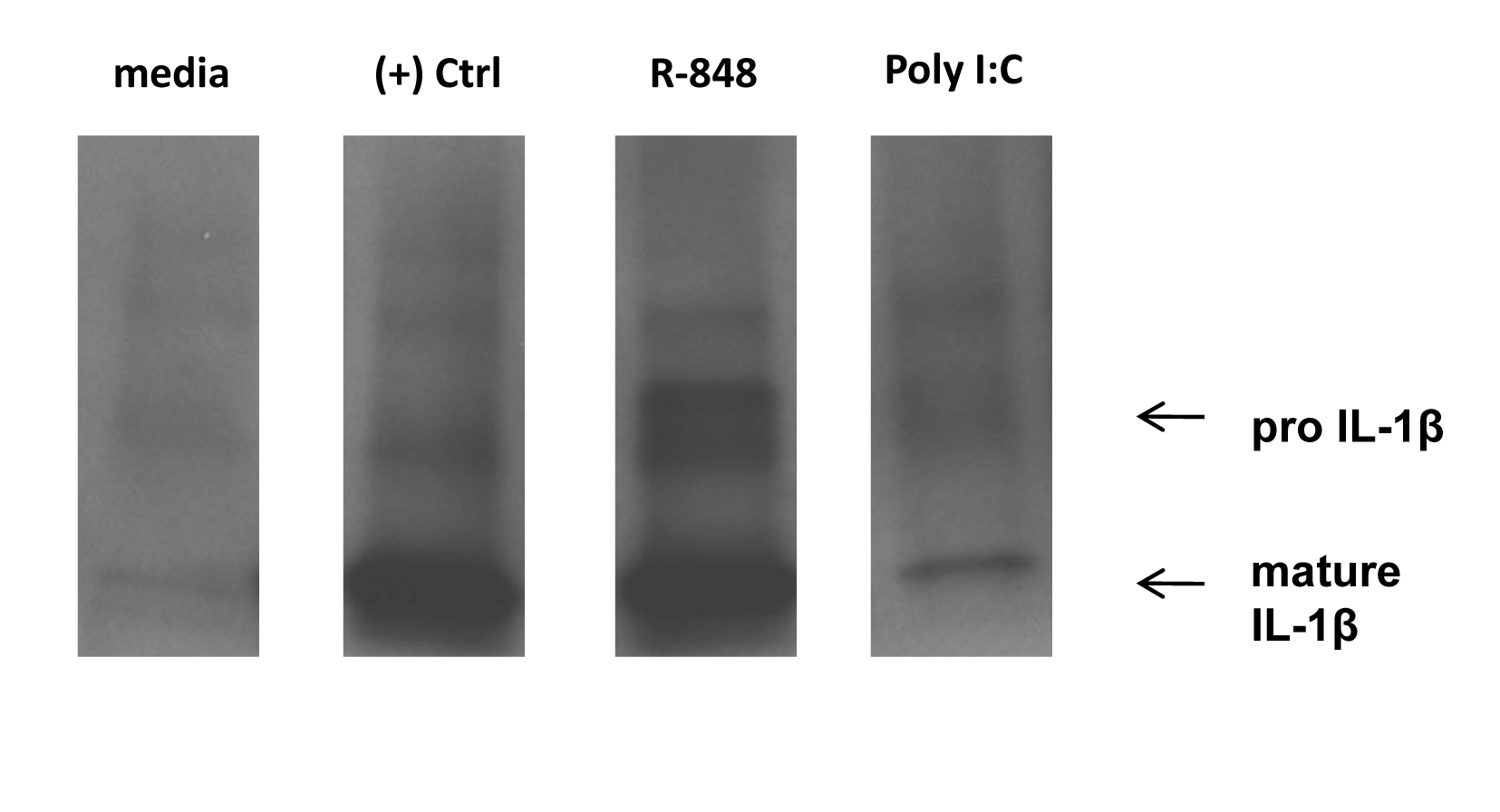

Supplement: Figure S1 — R-848 and extracellular ATP stimulates early mature IL-1β secretion from cDCs. Non-primed DCs were stimulated for 6 h with R-848 or poly I:C with extracellular ATP (5 mM). As a positive control, cDCs were primed with lipopolysaccharide (200 ng/ml) for 3 hours prior to stimulation with nigericin (10 µM). Mature IL-1β protein was detected by Western blot in cell culture supernatants. Data shown is representative of three independent experiments. (TIF) [file pone.0029412.s001.tif]
